# Meet the authors: Yuxuan Zhao, Enmeng Lu, and Yi Zeng

**DOI:** 10.1016/j.patter.2023.100891

**Published:** 2023-12-08

**Authors:** Yuxuan Zhao, Enmeng Lu, Yi Zeng

**Affiliations:** 1 Brain-inspired Cognitive Intelligence Lab, Institute of Automation, Chinese Academy of Sciences, Beijing 100190, China; 2 Center for Excellence in Brain Science and Intelligence Technology, Chinese Academy of Sciences, Shanghai 200031, China; 3 School of Future Technology, University of Chinese Academy of Sciences, Beijing 100049, China; 4 School of Artificial Intelligence, University of Chinese Academy of Sciences, Beijing 100049, China; 5 Center for Long-term Artificial Intelligence, Beijing, China

## Abstract

Yuxuan Zhao, associate professor, Enmeng Lu, research engineer, and Yi Zeng, professor and lab director, have proposed a brain-inspired bodily self-perception model based on biological findings on monkeys and humans. This model can reproduce various rubber hand illusion (RHI) experiments, which helps reveal the RHI’s computational and biological mechanisms. They talk about their view of data science and research plans for brain-inspired robot self-modeling and ethical robots.

## Main text

**Figure:**
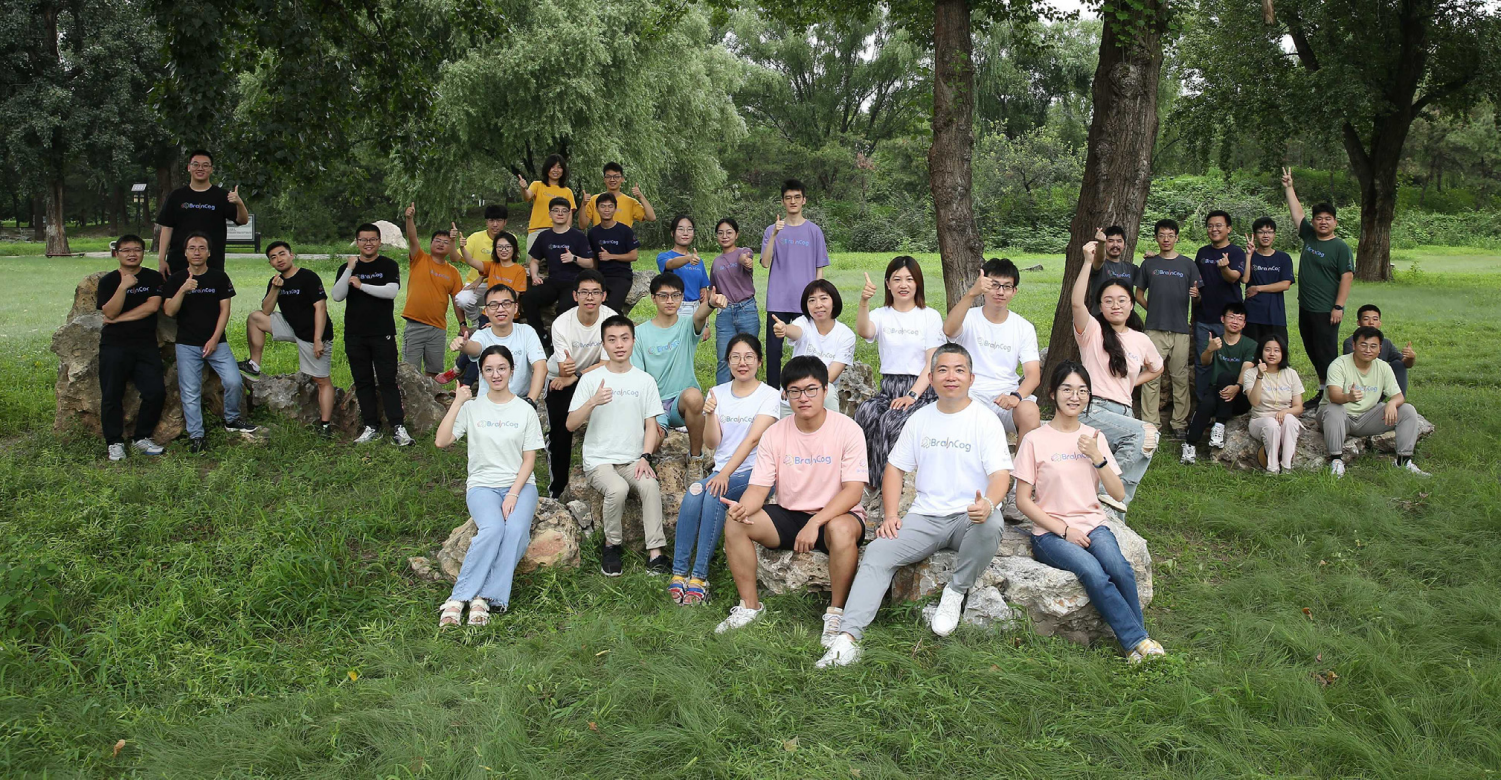
Group photo of Brain-inspired Cognitive Intelligence Lab (Yuxuan Zhao, leftmost first; Enmeng Lu, last row, fifth from the right; Yi Zeng, first row, second from the right.)

This interview is a companion to these authors’ recent paper, “Brain-inspired bodily self-perception model for robot rubber hand illusion.”^1^

### What would you like to share about your background?

**Yuxuan Zhao:** My first degree was a bachelor's degree in pharmaceutical engineering. During my 4-year undergraduate education, I discovered my passion for computer science and began self-studying in the field. My formal journey into computer science research began in 2013 at Peking University, where I subsequently earned a master's degree in software engineering, followed by a doctor of engineering degree in electronics and information. Afterward, I joined the Brain-Inspired Cognitive Intelligence Lab to engage in research on brain-inspired AI. My research focuses on brain-inspired cognitive computation models, higher cognitive function simulation, and brain-inspired cognitive robotics. We design brain-inspired cognitive computation models based on knowledge from various disciplines, including brain science, cognitive psychology, and neuroscience, to simulate higher cognitive functions. We explore the computational and biological mechanisms of higher cognitive functions from a computational perspective. My ultimate goal is to construct a robot self-model with self-consciousness. On one hand, to explore the biological mechanisms behind self-consciousness, and on the other hand, to enable the robot to make behavioral decisions that align with human values and ethics.

**Enmeng Lu:** My initial areas of research were in robotics and embedded systems design. Over time, I have gradually shifted my focus to AI, exploring how to build smarter "brains" for robots, and further, I have begun to think about how smarter robots in the future can coexist safely and ethically with humans. My engineering background has equipped me with a robotic perspective on embodied intelligence, while my longstanding interest in AI safety and ethics has led me to recognize the urgency and necessity of building safer and more ethical robots for the future. In pursuit of this goal, I am committed to building brain-inspired embodied cognitive intelligence implementations that empower future robots to be both more "self-aware" and more "empathetic towards others."

**Yi Zeng:** Motivated by sci-fi movies on AI when I was a bachelor student, I discovered my scientific vision as building AIs that can live symbiotically with humanity and obtained my PhD in AI. Later on, I joined the Institute of Automation, Chinese Academy of Sciences, where I established the Brain-Inspired Cognitive Intelligence Lab and the International Research Center for AI Ethics and Governance. I also founded the Center for Long-term AI to investigate long-term and societal challenges of AI for human and ecological good. I am generally interested in brain-inspired AI, AI ethics, safety, and governance, and AI for sustainable development. My long-term goal is to develop comprehensive theories and systems to decode the mechanisms and principles of human intelligence and its evolution and to develop artificial brains for brain- and mind-inspired conscious living AIs with or beyond human-level intelligence for a future human-AI symbiotic society.

### What motivated you to become a researcher? What helped guide you on your path?

**Yuxuan Zhao:** My motivation to become an AI researcher stems from my passion for knowledge and exploration. I am inherently curious about the unknown, and I have a deep desire to uncover the essence and truths of the world. I hope that through my efforts, I can contribute to humanity's understanding of the world. On my path to becoming an AI researcher, the team leader Professor Yi Zeng had a profound impact on me. He not only provided the position but also helped me establish a solid foundation and guide me towards the path of success with his cutting-edge academic thinking and passion for research. His ideas inspired me to delve deeper into the subject and encouraged me to challenge traditional ways of thinking. He always gives me ample freedom and resources to explore my research interests and provides selfless help and guidance when I encounter difficulties. In summary, it is my interest, passion, and the influence of those around me that have led me to embark on the path of AI research and continue to explore and grow in this field.

**Enmeng Lu:** I think sci-fi movies played an important role in defining and shaping my research career. During my student years, films like *Artificial Intelligence* and *Bicentennial Man*, which depict a future where humans and robots coexist harmoniously, fueled my aspirations and made me eager to work towards realizing such a future. On the other hand, scenes of robots losing control in movies like *2001: A Space Odyssey* and *I, Robot* prompted me to contemplate how to ensure that interactions between humans and robots in the future will always remain safe and controllable. While delving into robotics research, I realized that the actual state of the field was quite distinct from what I had seen in movies. Nevertheless, I believe that the initial visions and thoughts inspired by these films had planted the indispensable seeds for my research career.

**Yi Zeng:** In Steven Spielberg's 2001 film *Artificial Intelligence*, two researchers were discussing the simulation of the human brain in order to build robots that can love. This scene inspired my research on brain-inspired AI when I was in college. I realized that this is what I want to do with my life. That is, to build robots that can love humans. Based on these inspirations and motivations, I have been working for more than a decade to build “brain and mind inspired AI”—artificial intelligence that are inspired by the structure and mechanisms of the human brain and mind and could morally interact and co-exist with humans.

### Which of the current trends in data science seem most interesting to you? In your opinion, what are the most pressing questions for the data science community?

**Yi Zeng:** I am very interested in how we can develop brain- and mind-inspired ethical AI-incorporating efforts from data science and AI models. Much progress in AI, such as generative AI, has created more challenges than our society can solve in time. Since current AI is still an information-processing tool with many safety and security risks, ill design, misuse, and abuse may pose catastrophic risks for humans. Effective technical and social mechanisms need to be implemented to achieve ethical and safe AI. I am devoted to lead my team to work on how we can build brain- and mind-inspired moral AI models supported by data from human development, evolution, and behavior.

### Regarding your opinions on AI and robotic ethics, could you expand on how this relates to your research on the rubber hand illusion and bodily self-awareness?

**Enmeng Lu:** As we mentioned earlier, ethical robots of the future must first be controllable by humans, then be safe and ethical in their interactions, and finally, exhibit morality in their judgments and decision-making. Looking from the perspective of physical interaction in the future, robots need to learn not to cause physical harm to others (including themselves) by their actions and should also possess sufficient empathy when harm is occurring to others. For this future, robots must first have representations of their own bodies, distinguishing what belongs to them, others, and the environment, and need to have their “understanding” of what constitutes harm to their bodies, what “harm” itself implies, and what harm to others signifies. They should also predict which of their actions may cause harm and how to take measures to prevent it. We believe allowing robots to have human-like representations and perceptions of their bodily self, as well as adopting human-like ways of representing and understanding the notion of body and harm, is the basis for achieving these.

### How did this project you wrote about come to be?

**Yi Zeng:** In our Brain-Inspired Cognitive Intelligence Lab, we share a common understanding that self-perception is the basis for both natural and artificial intelligence. It is the foundation for self-consciousness and is essential for self-awareness, self-recognition, all the way to higher levels of cognition, such as learning and decision-making based on self-experience. Hence, computational models of self-consciousness and its inspiration to brain- and mind-inspired conscious AI is one of our ultimate goals. With several colleagues working together, we start with combining theoretical and data-driven approaches to build computational models of self-perception. The research of brain-inspired robot mirror self-recognition^2^ is our first try, and this time, we focus on brain-inspired bodily self-perception model for robot rubber hand illusion, which is even more fundamental compared to our research on our brain-inspired robot mirror self-recognition over 7 years ago.

### Was there something particular that motivated you to start or participate in this project?

**Yuxuan Zhao:** Building a bodily self-perception model for robots and attempting to reveal the underlying biological mechanisms of bodily self-consciousness from a computational modeling perspective is a very challenging task. We have chosen the rubber hand illusion experiment as an experimental paradigm to explore bodily self-consciousness. However, we lack biological experimental results to validate the model's biological plausibility and effectiveness. The experiments conducted by Fang et al.^3^ on macaques and humans have provided us with very important data and great inspiration for our work on AI. They provide objective and quantifiable behavioral experimental results as well as neuronal-scale experimental results, which can validate the effectiveness of our model at both the behavioral and neuronal scales. Building on this foundation, we have also selected some other rubber hand illusion experiments, including the less-studied Proprioceptive Precision experiment, and designed a disability experiment to further validate the model's efficacy. In addition to its contribution to AI, these results have the potential to assist researchers in cognitive psychology and cognitive neuroscience in further exploring the biological mechanisms of the rubber hand illusion.

**Enmeng Lu:** Yes. I remember a scene that stood out to me while watching the movie *Big Hero 6*. When Baymax, an inflatable healthcare robot, tries to get out of the main character Hiro's bedroom after it's been activated, it accidentally bumps off the books on the table, despite measuring its chubby body carefully down the aisle. This scene is a good example of the significance of endowing robots with a sense of bodily self-representation for their decision-making and safety concerning both themselves and their surroundings. This is especially pertinent in cases like Baymax, where the physical boundaries of the body change dynamically with inflation and deflation. It also serves as one of the starting points in our work to explore the concept of a robot's bodily self-awareness.

### What’s next for the project?

**Yuxuan Zhao:** The ultimate goal of the project is to construct a self-model for robots, which is an intriguing and challenging task. We will build the robot's self-model from five aspects: embodied self, agential self, spatiotemporal self, social self, and conceptual self. Fortunately, we have accumulated some research foundation in the preliminary phases. In terms of embodied self, we have developed a brain-inspired bodily self-perception model that allows robots to autonomously construct body models, induce rubber hand illusions,^1^ and pass mirror testing.^2^ In terms of agential self, we have developed a brain-inspired classical conditioning model^4^ that can reproduce as many as 15 classical experiments and enables robots to establish classical conditioning and acquire the ability for speed generalization. In terms of social self, we have developed a brain-inspired theory of mind model^5^ that allows robots to use their own experiences to infer the beliefs of other robots and predict the behavior of others. Next, based on existing work, our team and I will integrate new experimental paradigms and biological findings to develop cognitive models centered around the five aspects of self. This will allow us to continuously enrich and refine the robot's self-model. Furthermore, while exploring the biological mechanisms, we will also deploy the model to robots to investigate their applications in daily life.

### Prof. Zeng, what kind of atmosphere do you look to foster in your team? Is there anything you try to replicate or avoid from your own experiences or that you have learned over the years?

**Yi Zeng:** The atmosphere of our team is very free, cooperative, and harmonious. A phrase that I have summarized from my years of research and that I use to encourage my students is: Think creatively, think visionarily! Think differently, think big! Act small, start now!

### Aside from supervising their research, how do you help to develop and mentor your students and postdocs as scientists?

**Yi Zeng:** I focus on helping students and my staffs in both their academic tastes and their values as positive contributors to science and society. For example, to do meaningful and innovative research for the good of humanity, and to be upright, honest, ethical, and with great compassion. At the same time, it is important to have self-confidence, perseverance, and patience to do solid work.
